# The role of the State Security Service (Stasi) in the context of international clinical trials conducted by western pharmaceutical companies in Eastern Germany (1961–1990)

**DOI:** 10.1371/journal.pone.0195017

**Published:** 2018-04-02

**Authors:** Rainer Erices, Andreas Frewer, Antje Gumz

**Affiliations:** 1 Institute for History of Medicine and Medical Ethics at the University of Erlangen-Nuremberg, Erlangen, Germany; 2 Berlin University of Psychology (PHB), Berlin, Germany; 3 Department of Psychosomatic Medicine and Psychotherapy, University Medical Center, Hamburg-Eppendorf and Schön Klinik Hamburg Eilbek, Hamburg, Germany; Mahidol-Oxford Tropical Medicine Research Unit, THAILAND

## Abstract

**Background:**

After the building of the Berlin Wall in the 1960s, a number of international pharmaceutical manufacturers from the West had their drugs tested in Eastern Germany (GDR). So far, the extensive collection of documents on the subject stored in the archives of the GDR State Security Service (Stasi, MfS) has not been systematically analysed. Until now, the role of the Stasi with respect to the surveillance of the trials has been unclear.

**Methods:**

A keyword search within the database of the Stasi files was conducted. All available files were screened in order to identify institutions, companies and personnel involved in the clinical trials. On this basis, further files were requested. A total of 259 files were available for analysis. Relevant data was derived from 160 of these files. A contextualised approach was applied, which critically explored the origin, content, and impact of the data. In addition, an approach guided by the central steps of document analysis was applied.

**Results:**

At least 400 clinical trials were conducted during the GDR period. The exact number remains speculative. According to references found in the Stasi files, it might have been considerably higher. Initially, the main goal of the trials was for the GDR authorities to decide whether to import certain Western drugs. By 1983, this intention had changed. Now, the primary aim of the trials was the procurement of foreign currency. The Stasi feared that the pharmaceutical companies could have a significant influence on GDR Health System. Stasi spies were holding positions in the responsible medical committees, universities, and hospitals. Constant surveillance by the Stasi served the purpose of monitoring any contact between people from the West and the East. Unknowingly, representatives of Western companies were surveilled by the Stasi. The studied documents also point to the fact that a number of clinical trials conducted during the GDR period did not comply with GDR regulations, and were therefore deemed illegal by the Stasi. The Stasi was not particularly interested in medico-ethical questions.

**Conclusions:**

Clinical trials conducted during the GDR period were surveilled by the Stasi. It was their aim to monitor all people involved in the trials, including their Western contacts. Relevant medico-ethical questions like patient consent and safety with respect to the clinical trials were not the focus. Considering the significant number of conducted trials, only limited evidence exists of doctors having discussed them critically. The public was not officially informed about the trials.

## Introduction

Over the last few years, clinical trials carried out by Western pharmaceutical companies in the German Democratic Republic (GDR) have gathered a great deal of media attention. A first scientific publication on the topic based its findings on documents from the archives of the GDR Ministry of Health. [1] This study showed that 220 clinical trials with more than 14,000 patients were conducted between 1983 and 1990. A comprehensive study by Hess et al. (2016) reported the occurrence of 300 trials within the same time period. [2] The documents studied so far do not give a conclusive answer to whether all participants consented to participate in the trials. The GDR did not have standardised patient consent forms and/or information sheets. [1, 3, 4] However, documents indicate that patient consent depended on whether Western pharmaceutical companies insisted on it. [2] In general, the GDR population was not informed about the trials. [1]

The clinical trials were carried out during a phase in which the GDR was economically weak. [5] On the world market, the country’s own currency was effectively worthless. As a result, the state’s health sector was obliged to generate urgently needed hard currency. [1, 6, 7] In GDR files, requests for products that could profitably generate foreign currency can repeatedly be found. [6, 8] The need for foreign currency increased immensely at the beginning of the 1970s. One reason for this was the consumer friendly politics of the GDR head of state, Erich Honecker. [9] The GDR had to take out loans with overseas banks in order to obtain the foreign currency needed for imports. Towards the end of the 1970s, at least ¾ of all imports were financed through loans. At the time, the indebtedness to the West already amounted to more than 20 billion DM. This situation was aggravated in the 1980s, especially since the interest rates were relatively high. [6]

Some countries of the Eastern bloc, such as Poland, Hungary or Romania became insolvent. Internationally operating banks were unwilling to pay out loans. In 1983, the GDR too was close to insolvency. In response, the government carried out a crisis management plan, which asked all areas of the economy to generate more foreign currency. [10] During that year, an additional 300–400 million German Mark (West) was supposed to flow into the treasury. Responding to the governmental demand for higher foreign currency revenues, the Ministry of Health developed four central pillars of special export services for the GDR health sector: The GDR sold blood and blood products to the West; foreign doctors were trained and patients were treated for hard currency. [11] In addition, drugs from Western manufacturers were systematically tested across the country.

**Fig 1:**
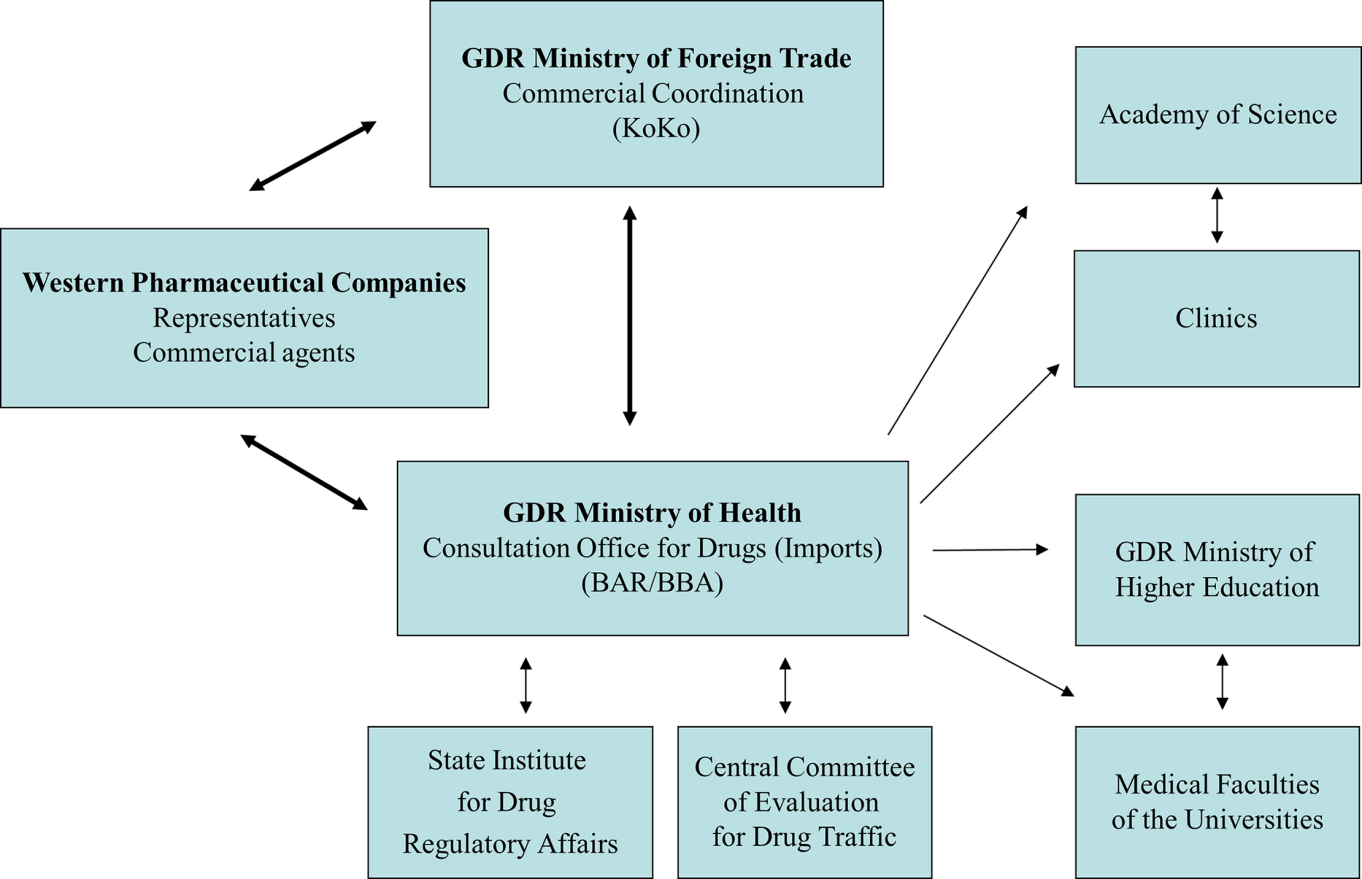
Relationship between institutions during clinical trials of Western pharmaceutical manufacturers. From 1983 onwards, the GDR Ministry of Health tried to centrally coordinate all clinical trials. Relevant contracts were drafted by the GDR Administration of Foreign Trade. The Institute for Drug Regulatory Affairs and the Central Committee of Experts were the subject-specific control organisms. The trials were conducted in clinical and academic institutions throughout the country belonging to different ministries and the Academy of Science.

These clinical trials were coordinated by the Ministry of Health. [12] In addition, several administrative departments of the GDR participated in the organisation of the trials (Fig 1). The Health authorities shared its control over the trial parameters with a special department of the Ministry of Foreign Trade, the "KoKo" (“Kommerzielle Koordinierung” = Commercial Coordination). [1] The KoKo worked closely with the Stasi [13] and was in charge of all financial and contractual agreements with Western pharmaceutical companies.

Generally, the historical reappraisal of the GDR health sector is still in the early stages. So far, mainly reviews on its development exist, as well as reviews on certain departments within the health sector, such as psychiatry. [14–17] In addition, numerous articles deal with the GDR doctors who worked as spies for the Stasi [11, 18–20] To date, only a few articles have addressed the pharmaceuticals market within the GDR. [21, 22] Articles on the clinical studies of Western manufacturers within the GDR are therefore an important step towards addressing this research gap.

In the post-reunification period already, the Berlin Medical Association appointed an investigative commission to look into public allegations that had been made concerning unwarranted clinical trials within the GDR. [23] Using patient files and witness’s statements, the commission concluded that these allegations were unsubstantiated. However, scandalising media reports have since highlighted the need for a systematic analysis of the issue.

The aim of the present article was to be the first to systematically analyse the role of the Stasi in the clinical trials, using accessible Stasi documents. The Ministry of State Security was one of the main forces that helped maintain the communist regime in the GDR. Like all parts of society, the health system was under constant secret surveillance by the Stasi [11]. The Stasi documents can therefore provide comprehensive information with respect to the clinical trials. Against this background our aim was to find answers to the following questions:

1. Which new insights do the Stasi files provide?
2. What reasons did the Stasi have to secretly surveil the clinical trials of Western pharmaceutical companies conducted in the GDR?
3. Did GDR doctors assist the Stasi in their surveillance of the clinical trials?
4. What medico-ethical evaluation do the newly investigated files suggest?

## Methods

### Search procedure and file selection process

A request to gain access to documents concerning the clinical drug trials was sent to the Federal Commissioner for the Stasi records (BStU). Upon demonstrating a research interest, files from the Stasi can be accessed by the public. Ours was the first comprehensive application for this research topic with the BStU. The Stasi files were selected using a typical step-by-step evaluation approach. In a first step, a keyword search within the BStU database ("clinical trial"; "drug tests"; "Western company"; "BBA") was conducted. This yielded a variety of files on clinical trials in the GDR. Any patient information was anonymised and de-identified by the BStU administration prior to analysis.

All available files were screened by the authors in order to identify institutions, companies and personnel involved in the clinical trials (Fig 1). On this basis, further files were requested. This process yielded a large number of additional files. A total of 259 files were available for analysis, 65 of these contained several volumes. The files contained:

a) Reports, evaluations, statistical data, correspondence of the Stasi about the activity of Western pharmaceutical companies in the GDR,
b) Reports, analyses and verbal agreements between different GDR Ministries and the institutions relevant for the trials,
c) Confidential reports of so-called unofficial collaborators ("IM") and investigation files for doctors and employees of Western pharmaceutical firms ("OV").

Most of these files belonged to the main Stasi department XX/1, which was responsible for the GDR health service.

### Data analysis

Using a contextualised approach, we critically explored the origin and content of specific archival and published textual material. This approach was accompanied by the central steps of document analysis [24]. Document analysis involves skimming (superficial examination), reading (thorough examination), and interpretation. [25] Firstly, while reviewing the documents, we identified meaningful and relevant text passages. Secondly, we allocated these text passages to nine thematic codes, which were developed based on previous research of our research group. [1] Please refer to supporting information S1 Table for an overview of how the codes relate to our research questions. Relevant data was derived from 160 of the 259 files (S1 Text). The subsequent thematic analysis involved a careful, more focused re-reading and review of the data. We took a closer look at the selected files to gather information pertinent to the central research questions.

The analysis of Stasi files is fundamentally different from the analysis of other primary sources. The Stasi collected material in a comprehensive manner (total surveillance). However, their aim was surveillance, not an in-depth examination of a topic. Furthermore, Stasi employees were no medical professionals. Nevertheless, the files are of particular relevance given that they do not only depict prominent people, institutions or events, but the entire society. From the GDR leaders’ fear of being undermined resulted an exhaustive surveillance apparatus of society, especially during the 1970s and the 1980s. [26] Today, the documents offer information on all forms of resistance, adaptation and participation with this particular political system.

Access to the files is partly limited by data protection regulations. These were noted down in the Stasi Files Law (i.e. “Stasi-Unterlagen-Gesetz”) of 1991, which was primarily passed to protect Stasi victims. In contrast to the public, researchers are granted relatively open access to the files.

A large proportion of the Stasi files deals with the relationship between East and West Germany. In line with the present study, one primary focus was on how the GDR monitored doctors or other medical personnel and their Western contacts. A vast amount of unofficial Stasi personnel made this surveillance possible. Spies amongst doctors were especially frequent. According to today’s estimates, 3–5% of the doctors were spies. [19]

We also compared our findings to material stored within the German Federal Archives (e.g., files from the GDR Health Ministry). [1, 27]

## Results

### 1. New insights drawn from the Stasi archives

The Stasi files show that between the building of the Berlin wall and 1990, the GDR conducted clinical trials for Western pharmaceutical companies on a regular basis. Two significant time periods can be singled out (Table 1). Before 1983, GDR doctors were trialling Western drugs with the purpose of evaluating their prospective use in the country. [28] Only a few files suggest that the GDR was running the trials on a commercial basis during this phase. The financial return for the trials was relatively low at that time. After 1983, however, the procurement of hard currency became the primary purpose. By that time, the trials were centrally organised and monitored. [1]

**Table 1:** Two significant time periods during the clinical trials of Western manufacturers in the GDR.

Time period	Number of clinical trials *	Number of patients involved	Number of Western firms involved	Primary aims
**1961–1982**	>187	Unclear	110	- Import - Purchase of a licence - Reproduction - Procurement of foreign currency
**1983–1990**	>220	>14,000	68	- Procurement of foreign currency

A classification of the tested drugs can be found in the Supporting Information (S2 Table). A list of the Western companies involved can be found in the Supporting Information (S3 Table).

*These numbers are minimum values. They are based on official figures from the GDR Health Ministry. Within the Stasi files, there are considerably more references to trials.

We found about 400 references to trials having been conducted prior to 1983. However, many of these references do not contain relevant details (e.g., origin or source of the data, trial dates, number of participants etc.). In particular, we could not find relevant contracts for this period. Furthermore, files appear to contain incorrect dates and some files list clinical trials of other socialist states. It appeared more accurate to report a reliable *minimum value* of the total number of trials. We therefore decided to base our analysis exclusively on official overviews of trials, which the Stasi received from the GDR Ministry of Health. These overviews contain information on trials that were running and that had been completed between 1973 and 1981. The available statistics show that at least 187 different drugs for 260 medical indications were tested during this period. A number of these trials were conducted without a contract, i.e. without having informed the Western manufacturer. [29]) Despite systematically searching through the Stasi files and Health Ministry files, official summaries from 1961 until 1983 could not be found. Therefore, the exact number of drug trials that were conducted before 1983 remains speculative.

After 1983, Stasi files provided hardly any new information regarding the types of conducted trials and the total number of conducted trials. The documents of the GDR Health Ministry, which are currently stored in the federal archives, are better suited for historical reappraisal. From 1983 onwards, the trials that earned foreign currency were numbered consecutively. For the most part, relevant contracts are available. Hess et al., for example, found references to more than 300 trials having been conducted in this time period. In their analysis, animal trials, medical technology trials, and follow-up studies were counted. Several trials were initiated but then stopped, and some trials were postponed and not conducted until the end of the GDR. To not overestimate the total number of conducted trials, we decided to rely the ones that were consecutively numbered (220 trials). Hence, our analysis is based on the minimum number of conducted trials. It represents a conservative estimate. This means that the total number of clinical trials conducted from 1961 to 1990 amounts to at least 400. More than one hundred Western companies took part in the trials. [1, 29] It can only be speculated how many patients were involved, but 20,000 seems to be a reasonable estimate.

From 1983 onwards, the majority of trials were organised centrally. [1] Files indicate that several clinical trials were conducted without official authorisation. [29–31]

Stasi files show that the GDR was a good market for the products of Western manufacturers. In 1988, an agent reports that "without exception all pharmaceutical companies" were interested in doing business with the East: the GDR was seen as a "pacemaker" for the licence and export business with the Eastern bloc. [32]

The Health authorities shared its control over the trials with a special department of the Ministry of Foreign Trade, the KoKo. It was entitled to half of the revenue in foreign currency. It was of no interest to the Koko that from a medico-ethical standpoint, the drug tests were a sensitive part of clinical investigation. [33]

From the 1980s onwards, some records show the beginnings of a critical debate with respect to the clinical trials. An undercover agent of the pharmaceutical industry stated that Western companies were especially interested in trials that could be carried out "particularly economically". This meant conducting trials that were comparatively cheap and that included a large number of patients. It also meant trials that "could no longer be carried out in the developed countries of the capitalist world, due to ethical or medico-scientific concerns". [33] In 1985, the research director of the Charité voiced concerns about the fact that the West only attempted to plan trials in the GDR, "which the Western press had already denounced as dishonourable, inhuman". [34] A Berlin professor and Stasi informant remarked that "manufacturers in Western Germany are facing more and more problems because the ethical committees in their country are practically preventing them from running clinical trials which could prove dangerous for the safety of the test persons." [35] The Stasi stated that companies were increasingly facing problems when trialling their substances in the West because "the local public opinion was strongly against this type of tests". [36] It claimed that the "slip of the tongue", calling GDR patients "guinea pigs", was "not completely removed from reality". At the time, the Hoechst affiliate Roussel Uclaf was testing the so-called abortion pill in the GDR ("*RU 486*"), despite vehement protests against the drug in Western Europe. This trial, however, was part of an international study. [2]

### 2. Surveillance by the Stasi and its goals

It was the primary aim of the Stasi to monitor whether Western firms had any influence on the GDR and, if necessary, to reduce this influence. The Stasi feared that contact with the West would bring "enemy ideologies" into the GDR and weaken the communist state. The representatives of Western firms and their correspondents in the GDR were the primary foci of surveillance. The Stasi suspected that these representatives were working as spies for the Western secret service. Furthermore, the Stasi assumed that it was their task either to spy out the results of East German scientific investigations, or to engage in "human trafficking", by offering help to GDR citizens who were planning to leave the country. [37–41]

In the 1960s and 70s, the Stasi collected more and more evidence that doctors contacted Western pharmaceutical companies without seeking official authorisation from the GDR Ministry of Health; therefore conducting clinical trials seemingly single-handedly. The Stasi was aware that hospital doctors were becoming frustrated with the constant shortage of medical materials and drugs. Many of them were trying to better the situation by applying methods from the West, even if it meant bypassing central admission procedures. [28, 42–45] The studied documents point to the fact that many clinical trials conducted during the first period (i.e. until 1983) did not comply with GDR regulations and were therefore deemed illegal by the Stasi. The term "illegal" refers to an assessment of the Stasi. All trials that were not centrally registered and/or organised were deemed "illegal". The term does not refer to an unethical execution of trials (i.e., medical aspects).

It appears that the Stasi was not particularly concerned about trials possibly violating GDR laws for clinical trials. It showed much more interest in political considerations. The Stasi feared that "pharmaceutical companies … were having [a massive influence ] on the GDR Health System". [46] At the end of the 1970s, the Stasi tried to limit Western companies operating in the GDR, in an effort to stop the "massive legal and illegal infiltration of the country with propaganda material …". [47] From then on, only state institutions served as negotiation partners for Western companies and only "politically trustworthy and specialized experts" were allowed to deal with the trials.

### 3. Doctors participating in the surveillance

In the leading positions of the Ministry of Health, the pharmaceutical industry, in medical faculties and clinics, the Stasi employed doctors and scientists as unofficial collaborators (IM), i.e., spies. A particularly high number of spies were working in the Consultation Office for Drugs (BAR/BBA), the central contact point for Western companies. Its directors and deputies had been Stasi spies for many years. The investigated files show that these persons had been recruited many years before. After having proved themselves faithful to state politics, they were put in leading positions. For years, they delivered comprehensive reports on representatives of Western companies and colleagues of their own team to the Stasi. [28, 33, 48] An important Stasi agent was the director of the General Department of Pharmacy and Medical Technology in the Ministry of Health, who was later promoted to State Secretary of Health. [49] Another reliable agent in a leading position was in charge of drug registrations and the cooperation with foreign companies. [45] Additionally, many doctors all over the country were working as Stasi spies. Some of them were renowned university professors of the country’s medical faculties. [50]

### 4. Medico-ethical assessment of the trials

The requirements for clinical trials in the GDR were almost identical to those in the West. Trial protocols indicated that all trials had to adhere to the guidelines of the Declaration of Helsinki, as well as GDR rules. There is no evidence in the Stasi files that patients’ rights were harmed intentionally or systematically during the trials. However, it is obvious that the Stasi was not particularly interested in medico-ethical correctness.

Evidence on critical discussions concerning the trials was found almost exclusively for the time after 1983. Due to the increasing demand for hard currency, the GDR agreed to conduct progressively more and larger trials for the West: Some of the doctors working as undercover spies started to criticise the high number of trials in GDR clinics. They also began questioning the patients´ safety in double-blind studies. Yet, the files do not contain any evidence of patients or personnel critically discussing clinical procedures, such as the necessity of informed consent. This is surprising considering the large number of trials and participants (e.g., patients and doctors). Doctors and scientists, as well as the public, were expected to subordinate their personal interests to the public ones. [51] In other words, state authorities prescribed that the interests of an individual corresponded to the interests of society. Independent views were not welcome in the GDR. Many years of manipulation had had the desired effect on the population, who suppressed its own voice to a remarkable extent. [52, 53] Most doctors accepted the existing state regulations. While patients in the West were becoming more self-confident and well informed, this phenomenon did not extend to the East. These conditions were favourable for Western firms. [1] In a country that had become completely dependent on foreign currency, no considerable resistance against clinical trials was to be expected. [54, 55] Added to this was the complete surveillance by an immense security apparatus, which protected the interests of the GDR-state. Western companies could generally rely on receiving results in the arranged (short) time period. The study costs could be calculated clearly. There was no danger of any form of public discourse. The GDR did not have much public awareness and therefore hardly any patients who were sceptical of pharmaceuticals. There was no freedom of press and therefore no potential disturbances were to be expected in case of an unplanned incident.

The fact that the public was not informed about the trials presents an important ethical problem: Patients were unaware that they could be administered unauthorised Western drugs during a hospital stay and thus, unknowingly, became participants in clinical trials.

## Discussion

The Stasi files provide new insights into the clinical testing of Western pharmaceuticals conducted in the GDR. Given the unsystematic file keeping, analysis and evaluation of the data proved difficult. The Stasi files on the clinical trials were collected as part of the general surveillance that was exercised by the GDR regime. The Stasi was not so much concerned with clinical or ethical questions concerning these trials.

The Stasi files show that as early as in the 1960s, a great number of trials were being carried out in the GDR. During these first years, doctors were mainly examining the possible use of certain drugs in the GDR. In the 1980s, the trials were particularly conducted as a means to procure foreign currency.

In the GDR, it was general practice to whitewash the socialist reality. [11] In the 1980s, the National Health System was close to breakdown. However, to this day, many ex-GDR citizens believe that it was one of the big achievements of the socialist society. [56] While state propaganda claimed "all for the good of the people", economical and ideological interests dominated day-to-day life. [57] A significant part of the revenues from clinical trials did not benefit the Health System but went to the secret accounts of the KoKo, a special department of GDR foreign trade, which was controlled by the Stasi. This shows that the focus was the procurement of foreign currency and not the patients´ well-being. In the GDR, decisions were made for ideological reasons. [58] State politics followed a socialist ideal. A collective code of ethics and ethical freedom was nonexistent. The rights of the individual were often secondary. [59] In many situations, the activities of doctors and medical staff were guided by the political interests of the state and not by professional ethos. [11, 19, 60]

From 1961 until 1990, the clinical trials were surveilled by the Stasi and as a result, enormous amounts of intelligence were gathered. With the help of undercover spies, the Stasi participated directly in the negotiations between West and East. Unofficial Stasi collaborators amongst doctors made it possible to keep the National Health Service under permanent observation. The large number of spies in all positions even remotely related to the clinical trials shows that they were of vital importance for the GDR. It shows that the trials were deemed to be of high risk for the political security of the state. It is important to mention that, whilst millions of GDR citizens conformed to the state by joining the socialist party, neither the general population nor doctors or scientists deemed an intimate personal relationship with the Stasi as morally acceptable. [18] The proportion of unofficial collaborators amongst employees of the GDR Health System was around 1%, with a considerably higher percentage amongst doctors, of about 3–5%. [19] One of the ethical standards of the Hippocratic Oath [61] states that any doctor has to show discretion towards their patient. It is the basis of a doctor’s position of trust and, as such, it is inconsistent with the tasks of an informant to a state security service. The national health system was subordinated to the political and ideological system of the GDR. In the close-knit structure of decision makers, head doctors and the Stasi were working hand in hand.

Files sporadically document doctors criticising the clinical trials and there is hardly any material on doctors discussing ethical questions. It can be assumed that noteworthy resistance to the trials would have been reflected in the files. Clearly, this was not the case amongst the doctors.

The requirements for pharmaceutical trials in the GDR were almost identical to those in the West. Trial protocols indicated that all trials had to adhere to the guidelines of the Declaration of Helsinki as well as the GDR rules. Still, Western companies had good reasons for conducting trials in the GDR: The GDR was dependent on foreign currency revenues and therefore likely to comply with contractual agreements. Test procedures were contractually regulated in advance and then centrally organized. The GDR Ministry of Health coordinated all processes relevant to the trials and supervised the implementation. Even in case of harm being done to patients, companies were contractually protected. Given the economic shortages, patients and personnel trusted in Western products. Furthermore, personnel hoped for material and/or personal advantages when collaborating with Western companies and monitors. An additional advantage for Western companies was the complete surveillance by an immense security apparatus, which protected the interests of the GDR-state.

How difficult an ethical assessment of a trial can be–especially when considering the particular conditions of the authoritarian GDR-state–is demonstrated by multi centre studies that were conducted solely in the GDR. As an example, we would like to refer to the *ramipril*-study by Hoechst, which was conducted with high-risk patients (I-107, placebo-controlled, double-blind, 12 centers, from 1987 onwards, 144 patients, 3,500 DM per case). [31, 33, 35, 62] According to Stasi information, this study led to discussions among doctors, given that it was conducted with patients with a high mortality risk. Fourteen patients died during the trial, most of which had been allocated to the verum group. [2] However, contrary to current speculations in the media, the files give no indication that the medication had caused the fatalities. Also: an ACE inhibitor (i.e., a pharmaceutical compound comparable to *ramipril*) was only being introduced at that time and therefore not a medical standard. Therefore, the placebo group received only treatment as usual. The verum group received the new product, which is one of the most important drugs for the treatment of hypertension and congestive heart failure today. According to Hess et al., company archives provide no evidence that Hoechst took advantage of the shortage of modern drugs in the GDR at that time. [2]

### Limitations

A common difficulty when working with Stasi files is the large quantity–the Stasi gathered information on every aspect of society. A noteworthy limitation of the study is due to the fact that the Stasi gathered information in an unsystematic manner. Therefore, the quality of the sources is somewhat compromised. In general, Stasi documents cannot be regarded as precise, accurate, or complete. They were produced for a purpose other than research. Generally, the files contained extremely detailed, yet general reports. However, they do give a good insight into of the clinical trials conducted in the GDR.

Additionally, it is necessary to maintain a certain distance to the material and to put the information into context. Interpreting the information with a critical eye was especially important in the context of the issue under investigation. In contrast to the whitewashed depiction of issues in GDR society, the aim of the Stasi was to document reality. However, it has to be taken into account that the Stasi also saw reality through an ideological lens. Despite this, contemporary historical research regards the Stasi files as important sources for the historical reappraisal of the GDR period. [63]

## Conclusion

The Stasi files offer new information on the clinical trials that were conducted in the GDR. In total, more than 400 clinical trials were documented. Initially, the main goal of the trials was for GDR authorities to decide whether to import certain Western drugs into the country. By 1983, this intention had changed; the trials were mainly conducted to procure foreign currency. The Stasi monitored the clinical trials in order to have control over and to reduce Western influences on the GDR health sector. Since there were many Stasi spies among the doctors and those in charge, a vast amount of information was accumulated on the trials.

The Stasi was only marginally interested in ethical questions like patient consent and safety.

In terms of security policy, it served as a surveillance apparatus. The primary aim of the Stasi was to monitor and control doctors and their Western contacts. Medico-ethical questions, which are of importance in clinical drug trials, were not the focus of their investigations. Nevertheless, answers to these questions can be found within the Stasi files. The Stasi collected information on all areas of society, including moods and opinions of everyone concerned. There is sporadic evidence about a critical debate on the trials amongst the doctors involved. However, considering the significant number of trials carried out and the constant surveillance by the Stasi, this number appears low. Furthermore, the public was not officially informed about the trials.

Western pharmaceutical manufacturers exploited the economic conditions of the GDR to conduct clinical trials. They benefited from the state’s immense economic dependency on the West. They were guaranteed comprehensive state control of their clinical trials–but in a foreign country. Even after the end of the Cold War, this topic is still relevant today. Given the progressive globalization in the healthcare industry and the pressure for cost reductions that companies face, the discussed problems are highly topical.

## Supporting information

S1 Table Initial codes and their relation to research questions. (PDF)

S2 Table Classification of the drugs tested according to ATC. (PDF)

S3 Table List of Western companies involved in the clinical trials in the GDR. (PDF)

S1 Text List of Stasi files reviewed. (PDF)
