# Supplementary material for: The role of the State Security Service (Stasi) in the context of international clinical trials conducted by western pharmaceutical companies in Eastern Germany (1961–1990)
Source: PLoS One. 2018 Apr 2;13(4):e0195017. doi: 10.1371/journal.pone.0195017 (PMC5880395; doi:10.1371/journal.pone.0195017)
Supplement: S1 Table — (PDF) [file pone.0195017.s001.pdf]

S1 Table

Initial codes and their relation to research questions

Initial codes		Research questions
I	Time. When was/were the trial/trials conducted?	1
II	Aims. What was/were the aim/aims of the trial(s)?	1, 2
III	Consent. What did patients know about tests?	1, 2, 3
IV	Role of Institutions involved (in German: BAR/BBA, ZGA, IfAR, clinics, Ministry of Health) and their employees	1, 2
V	Financial aspects and role of Ministry of Foreign Trade, "KoKo" department	1, 2
VI	Illegal drug tests	1, 2, 3
VII	Observation of Western companies and their employees	2, 3
VIII	Information on doctors, medical and administrative staff involved	2, 3
IX	Internal Stasi assessment, political and ethical aspects	1, 2

1=research question 1"Which new insights do the documents of the MfS provide?", 2=research question 2" What were the reasons of the MfS to keep clinical trials of Western pharmaceutical firms in the GDR under secret observation?", 3= Research question 3" How were GDR doctors involved in the Stasi observations?"
