# Supplementary material for: The role of the State Security Service (Stasi) in the context of international clinical trials conducted by western pharmaceutical companies in Eastern Germany (1961–1990)
Source: PLoS One. 2018 Apr 2;13(4):e0195017. doi: 10.1371/journal.pone.0195017 (PMC5880395; doi:10.1371/journal.pone.0195017)
Supplement: S2 Table — (PDF) [file pone.0195017.s002.pdf]

S2 Table

Classification of the drugs tested according to ATC-Codes

**Period**	**1960-1983**	**1983-1990**
**ATC-Code**		
A Alimentary tract, metabolism	25	27
B Blood, bloodforming	11	25
C Cardiovascular	20	43
D Dermatologicals	9	8
G Genito-urinary system, sex hormones	19	9
H Hormones, excl. sex hormones and insulins	6	9
J Antiinfectives	16	20
L Antineoplastic and immunomodulating agents	11	12
M Musculo-skeletal system	4	4
N Nervous system	22	26
P Antiparasitic products	1	0
R Respiratory system	11	16
S Sensory organs	3	0
V Various	29	21
Total	**187**	**220**
