# Supplementary material for: The role of the State Security Service (Stasi) in the context of international clinical trials conducted by western pharmaceutical companies in Eastern Germany (1961–1990)
Source: PLoS One. 2018 Apr 2;13(4):e0195017. doi: 10.1371/journal.pone.0195017 (PMC5880395; doi:10.1371/journal.pone.0195017)
Supplement: S3 Table — (PDF) [file pone.0195017.s003.pdf]

S3 Table

List of Western companies involved in the drug trials in the GDR

Period 1, 1960-1982
(187 trials)

	**Western company**
**Number of trials**	
**9**	Roche
**8**	Bayer
**6**	Boehringer-Ingelheim
**5**	Chemiewerk Homburg, Fresenius, Upjohn, Sandoz
**4**	B. Braun, SK&F, Janssen, Pfizer
**3**	Bristol Myers, Beecham, Byk Gulden, ICI, Pfrimmer, Schering, Knoll
**2**	3M, Abbott, MSD, Pharmachim, Astra, Ethicon, Nyegaard, Immuno, Kabi, Temca, Lipha Pharma, Merck, Squibb, Hoechst, Schaper & Brümmer, Leo Pharma, KRKA/Labaz, Eli Lilly, Zyma, Searle
**1**	Angelini, Asid Bonz, , Behringwerke, Beiersdorf, Bene-Chemie, Boehringer Mannheim, Boots, Bracco, Byk-Essex, Cambrian Chemie, Chemie Linz, Ciba-Geigy, Dansac, Dome, Dow Corning, Duphar, Farco-Pharma, Farmos, Fisons, Geistlich, Gerot, Gist-Brocades, Glaxo, Gore, Gry Pharma, Hartmann, Heyer Schulte, Hefa-Frenon, Hek/Roche/Dr. Falk, Henkel, Henning, Hermal-Chemie, Heyer, Huhtamäki, Inpharzam, Institut Bern, Jodogawa, , Kreussler, OM Pharma, Debat Laboratories, Lab. Sauba, Lab. Solac Toulouse, Lederle, , Lepetit, Linssi Oy, Mack, Madaus, Medimpex BRD, Merz & Dade, Mucos, Newpoint, Nordmark Werke, Novo-Nordisk, Organon, Paines, Parke-Davis, Pentapharm, Pharm-Allergan, Pharmacia, Pharmaton, Promonta, Robapharm, Roger Bellon, Solco, SRK, Temmler, UCB, Unipoint, Wellcome, Winthrop, Yoshitomi

Period 2, 1983-1990
(220 trials)

	**Western company**
**Number of trials**	
**32**	Boehringer Mannheim
**18**	Sandoz, Schering
**16**	Hoechst
**12**	Bayer
**10**	Ciba Geigy
**6**	Gödecke
**5**	Behringwerke, Braun Melsungen
**4**	Beecham Wülfing, Serono, Thiemann
**3**	Biotest, Cassella, Essex, Knoll, Midy, Orion, Upjohn, Syntex
**2**	Blend-a-med, Chemie Linz, DuPont, Espe, Hefa-Frenon, Kali Chemie, Medica, Pfizer, Pfrimmer, Roche, Röhm Pharma, Roussel Uclaf, Searle, SmithKline/Dome
**1**	Artesan, Astra, Bego Bremer Goldschlägerei, Boehringer Ingelheim, Byk Gulden, Cilag, Delagrange, Dr. Lenhard, Dr. Rentschler, Gambro, Gist-Brocades, Hennig, Homburg, LAB, Leiras, Leopold, Lomapharm, Madaus, Mead Johnson, Merck, Nattermann, Nordisk, Novo Mainz, Parke Davis, Pharmalog, Schütz Dental, Star, Strohschein, Thomae, Tropon, Verein f Krebsforsch, Verla-Pharm, Viradent, Voco-Chemie
