# Supplementary material for: The role of the State Security Service (Stasi) in the context of international clinical trials conducted by western pharmaceutical companies in Eastern Germany (1961–1990)
Source: PLoS One. 2018 Apr 2;13(4):e0195017. doi: 10.1371/journal.pone.0195017 (PMC5880395; doi:10.1371/journal.pone.0195017)
Supplement: S1 Text — (PDF) [file pone.0195017.s004.pdf]

**S1 Text**

**List of Stasi files reviewed**

BStU, MfS, AG BKK, Nr. 71
BStU, MfS, AG BKK, Nr. 163
BStU, MfS, AG BKK, Nr. 261
BStU, MfS, AG BKK, Nr. 263
BStU, MfS, AG BKK, Nr. 327
BStU, MfS, AG BKK, Nr. 348
BStU, MfS, AG BKK, Nr. 403
BStU, MfS, AG BKK, Nr. 759
BStU, MfS, AG BKK, Nr. 937
BStU, MfS, AG BKK, Nr. 1019
BStU, MfS, AG BKK, Nr. 1346
BStU, MfS, AG BKK, Nr. 1622
BStU, MfS, AG BKK, Nr. 2040

BStU, MfS, AGMS, Nr. 2104/81
BStU, MfS, AGMS, Nr. 5104/90
BStU, MfS, AGMS, Nr. 16867/89

BStU, MfS, AIM, Nr. 1245/87
BStU, MfS, AIM, Nr. 1407/91
BStU, MfS, AIM, Nr. 5668/76
BStU, MfS, AIM, Nr. 6043/79
BStU, MfS, AIM, Nr. 7405/91
BStU, MfS, AIM, Nr. 8117/91
BStU, MfS, AIM, Nr. 8150/91
BStU, MfS, AIM, Nr. 8276/91
BStU, MfS, AIM, Nr. 9125/91
BStU, MfS, AIM, Nr. 11029/91
BStU, MfS, AIM, Nr. 12750/83
BStU, MfS, AIM, Nr. 19794/63

BStU, MfS, AKG, Nr. 5863

BStU, MfS, AKK, Nr. 15072/85

BStU, MfS, AOP, Nr. 8165/78
BStU, MfS, AOPK, Nr. 13773/83

BStU, MfS, AP, Nr. 10632/85
BStU, MfS, AP, Nr. 12613/92
BStU, MfS, AP, Nr. 30727/92
BStU, MfS, AP, Nr. 35733/92

BStU, MfS, BV Berlin, Abt. XX, Nr. 2789
BStU, MfS, BV Berlin, Abt. XX, Nr. 2869
BStU, MfS, BV Berlin, Abt. XX, Nr. 3563
BStU, MfS, BV Berlin, Abt. XX, Nr. 7940

BStU, MfS, BV Berlin, AIM, Nr. 627/89
BStU, MfS, BV Berlin, AIM, Nr. 5572/91
BStU, MfS, BV Berlin, AIM, Nr. 6010/91
BStU, MfS, BV Berlin, AIM, Nr. 6105/91
BStU, MfS, BV Berlin, AGMS, Nr. 4314/91

BStU, MfS, BV Cottbus, AKG, Nr. 324

BStU, MfS, BV Dresden, AIM, Nr. 2248/84
BStU, MfS, BV Dresden, KD Dresden-Land, Nr. 15490
BStU, MfS, BV Dresden, KD Dresden-Land, Nr. 90092

BStU, MfS, BV Frankfurt/O, Abt. XX, Nr. 269
BStU, MfS, BV Frankfurt/O, Abt. XX, Nr. 805
BStU, MfS, BV Frankfurt/O, Abt. XX, Nr. 1661
BStU, MfS, BV Frankfurt/O, KD Frankfurt/O, Nr. V/1101/74, F16
BStU, MfS, BV Frankfurt/O, KD Frankfurt/O, Nr. V/1101/74, F22

BStU, MfS, BV Gera, Abt. XV, Nr. 1944
BStU, MfS, BV Gera, Abt. XVIII, Nr. 45/27
BStU, MfS, BV Gera, Abt. XVIII, Nr. 45/27 113
BStU, MfS, BV Gera, AGMS, Nr. 1097/89
BStU, MfS, BV Gera, KD Jena, Nr. X/60/84

BStU, MfS, BV Halle, Abt. XVIII, Nr. 2817
BStU, MfS, BV Halle, Abt. XVIII, Sach, Nr. 4676
BStU, MfS, BV Halle, Abt. XX, Sach 4127
BStU, MfS, BV Halle, Abt. XX, ZMA, Nr. 1110
BStU, MfS, BV Halle, AKG, Nr. 1830
BStU, MfS, BV Halle, AOP, Nr. 97/98
BStU, MfS, BV Halle, KD Bernburg, Abt. VIII, Nr. 843/77
BStU, MfS, BV Halle, KD Bernburg, Nr. 412
BStU, MfS, BV Halle, KD Roßlau, Sach 556
BStU, MfS, BV Halle, KD Roßlau, Sach 554
BStU, MfS, BV Halle, Nr. 207/65

BStU, MfS, BV Leipzig, Abt. XV, 984
BStU, MfS, BV Leipzig, Abt. XV, Nr. 1002
BStU, MfS, BV Leipzig, Abt. XV, Nr. 1979
BStU, MfS, BV Leipzig, Abt. XX, Nr. 1/05
BStU, MfS, BV Leipzig, Abt. XX, Nr. 208/03
BStU, MfS, BV Leipzig, Abt. XX, Nr. 246/01
BStU, MfS, BV Leipzig, Abt. XX, Nr. 821
BStU, MfS, BV Leipzig, AKG, 1922
BStU, MfS, BV Leipzig, KD Leipzig-Stadt, Nr. 491
BStU, MfS, BV Leipzig, Leitung, Nr. 894/04

BStU, MfS, BV Karl-Marx-Stadt, Abt. XIV, Nr. 921/77

BStU, MfS, BV Magdeburg, Abt. XII, AIM, Nr. 259/69
BStU, MfS, BV Magdeburg, Abt. XX, Nr. 1097

BStU, MfS, BV Neubrandenburg, KD Neubrandenburg, AGMS, Nr. XV/4281/81

BStU, MfS, BV Rostock, AGMS, Nr. 2528/82
BStU, MfS, BV Rostock, AIM, Nr. 2247/80
BStU, MfS, BV Rostock, AIM, Nr. 2929/87
BStU, MfS, BV Rostock, AIM, Nr. 4220/90
BStU, MfS, BV Rostock, AOPK, Nr. 2553/77
BStU, MfS, BV Rostock, KD Greifswald, ZMA, Nr. 3094
BStU, MfS, BV Rostock, Nr. 265/91

BStU, MfS, HA VIII, Nr. 2775
BStU, MfS, HA VIII, RF 1774/25, Nr. 13369/84
BStU, MfS, HA XVIII, Nr. 8911
BStU, MfS, HA XVIII, Nr. 15691
BStU, MfS, HA XVIII, Nr. 16214
BStU, MfS, HA XVIII, Nr. 20108
BStU, MfS, HA XVIII, Nr. 21171
BStU, MfS, HA XVIII, Nr. 21457

BStU, MfS, HA XX, AP, Nr. 40930/92
BStU, MfS, HA XX, AP, Nr. 71362/92
BStU, MfS, HA XX, Nr. 40
BStU, MfS, HA XX, Nr. 41
BStU, MfS, HA XX, Nr. 43
BStU, MfS, HA XX, Nr. 44
BStU, MfS, HA XX, Nr. 45
BStU, MfS, HA XX, Nr. 47
BStU, MfS, HA XX, Nr. 421
BStU, MfS, HA XX, Nr. 527
BStU, MfS, HA XX, Nr. 994
BStU, MfS, HA XX, Nr. 1466
BStU, MfS, HA XX, Nr. 1469
BStU, MfS, HA XX, Nr. 1572
BStU, MfS, HA XX, Nr. 1573
BStU, MfS, HA XX, Nr. 1903
BStU, MfS, HA XX, Nr. 2098
BStU, MfS, HA XX, Nr. 2940
BStU, MfS, HA XX, Nr. 5760
BStU, MfS, HA XX, Nr. 5765
BStU, MfS, HA XX, Nr. 6063
BStU, MfS, HA XX, Nr. 6542
BStU, MfS, HA XX, Nr. 6867
BStU, MfS, HA XX, Nr. 6668
BStU, MfS, HA XX, Nr. 6669
BStU, MfS, HA XX, Nr. 6670
BStU, MfS, HA XX, Nr. 6671
BStU, MfS, HA XX, Nr. 6843
BStU, MfS, HA XX, Nr. 6869
BStU, MfS, HA XX, Nr. 6898
BStU, MfS, HA XX, Nr. 6899

BStU, MfS, HA XX, Nr. 7084
BStU, MfS, HA XX, Nr. 7085
BStU, MfS, HA XX, Nr. 7150
BStU, MfS, HA XX, Nr. 7153
BStU, MfS, HA XX, Nr. 7201
BStU, MfS, HA XX, Nr. 7203
BStU, MfS, HA XX, Nr. 7209
BStU, MfS, HA XX, Nr. 7216
BStU, MfS, HA XX, Nr. 7218
BStU, MfS, HA XX, Nr. 7224
BStU, MfS, HA XX, Nr. 7244
BStU, MfS, HA XX, Nr. 7445
BStU, MfS, HA XX, Nr. 8302
BStU, MfS, HA XX, Nr. 10223
BStU, MfS, HA XX, Nr. 12277
BStU, MfS, HA XX, Nr. 12279
BStU, MfS, HA XX, Nr. 16559
BStU, MfS, HA XX/AKG, Nr. 115
BStU, MfS, HA XX/AKG, Nr. 1629
BStU, MfS, HA XX/AKG, Nr. 4254
BStU, MfS, HA XX/AKG, Nr. 5852
BStU, MfS, HA XX/AKG, Nr. 5853
BStU, MfS, HA XX/AKG, Nr. 6045
BStU, MfS, HA XX/ZMA, Nr. 5930

BStU, MfS, HA XXII, Nr. 735/16

BStU, MfS, HA XXVIII, Nr. 18515

BStU, MfS, Sekr. Mittig, Nr. 156

BStU, MfS, OPK, Nr. 4006/91

BStU, MfS, ZA, Allg., Nr. P 12646/70

BStU, MfS, ZAIG, Nr. 14492
